# Loss-induced nonreciprocity

**DOI:** 10.1038/s41377-021-00464-2

**Published:** 2021-02-04

**Authors:** Xinyao Huang, Cuicui Lu, Chao Liang, Honggeng Tao, Yong-Chun Liu

**Affiliations:** 1 State Key Laboratory of Low-Dimensional Quantum Physics, Department of Physics, Tsinghua University, Beijing, 100084 China; 2 Key Laboratory of Advanced Optoelectronic Quantum Architecture and Measurements of Ministry of Education, Beijing Key Laboratory of Nanophotonics and Ultrafine Optoelectronic Systems, School of Physics, Beijing Institute of Technology, Beijing, 100081 China; 3 Collaborative Innovation Center of Light Manipulations and Applications, Shandong Normal University, Jinan, 250358 China; 4 Frontier Science Center for Quantum Information, Beijing, 100084 China

**Keywords:** Nanophotonics and plasmonics, Microresonators, Photonic devices, Micro-optics

## Abstract

Nonreciprocity is important in both optical information processing and topological photonics studies. Conventional principles for realizing nonreciprocity rely on magnetic fields, spatiotemporal modulation, or nonlinearity. Here we propose a generic principle for generating nonreciprocity by taking advantage of energy loss, which is usually regarded as harmful. The loss in a resonance mode induces a phase lag, which is independent of the energy transmission direction. When multichannel lossy resonance modes are combined, the resulting interference gives rise to nonreciprocity, with different coupling strengths for the forward and backward directions, and unidirectional energy transmission. This study opens a new avenue for the design of nonreciprocal devices without stringent requirements.

## Introduction

Optical nonreciprocity, which prohibits a light field from returning along its original path after passing through an optical system in one direction, implying the breaking of the Lorentz reciprocity theorem, is crucially important for both fundamental studies and applied sciences^1–3^. For example, nonreciprocal devices, such as optical isolators^1^, optical circulators^4^, and directional amplifiers^5^, play important roles in optical communication and optical information processing. Moreover, the topological properties exhibited by nonreciprocal devices make them promising platforms for studying topological photonics^6,7^ and chiral quantum optics^8^. To date, a number of approaches have been suggested for generating nonreciprocity, including the use of parity-time (PT)-symmetric nonlinear cavities^9,10^, spinning resonators^11,12^, optomechanical interactions^13–20^, cavity magnonic interactions^21^, effective gauge fields^22,23^, and the thermal motion of hot atoms^24,25^. Despite these achievements, the basic principles for realizing optical nonreciprocity remain limited as a result of the time-reversal symmetry and linear nature of Maxwell’s equations. The existing approaches can be grouped into three categories with the following requirements^1–3^: (i) magnetic-field-induced breaking of time-reversal symmetry^26–29^, (ii) spatiotemporal modulation of system permittivity^30–36^, and (iii) nonlinearity^37,38^. However, these principles either encounter difficulties in integration^39^, require stringent experimental conditions^40^, or have limited performance^38^. Therefore, it is crucial to break the Lorentz reciprocity theorem by going beyond these approaches.

Here we devise a new principle for realizing optical nonreciprocity by making use of loss. Although it is obvious that loss breaks time-reversal symmetry, it is generally believed that loss cannot lead to optical nonreciprocity as a result of restricted time-reversal symmetry^2,3^, in which the field amplitudes are reduced while the field ratios are conserved. However, we show that loss under multiple channels with interference gives rise to optical nonreciprocity. The basic principle is that the phase lag induced by loss, which is independent of the energy propagation direction, results in different interference outcomes for the forward and backward directions. In our scheme, neither a magnetic field, the spatiotemporal modulation of permittivity nor nonlinearity is required. On the contrary, the resource we take advantage of is simply energy loss, which is regarded as harmful and undesirable in most studies. This is also different from PT-symmetric schemes in which the nonreciprocity originates from nonlinear gain with saturation. Our scheme is universal for a variety of physical systems, such as optical cavities and waveguides. This study paves the way for the observation of nonreciprocity and corresponding device design in lossy systems without stringent conditions, and provides opportunities for studying chiral and topological properties in systems with lossy coupling.

## Results

**Fig. 1:**
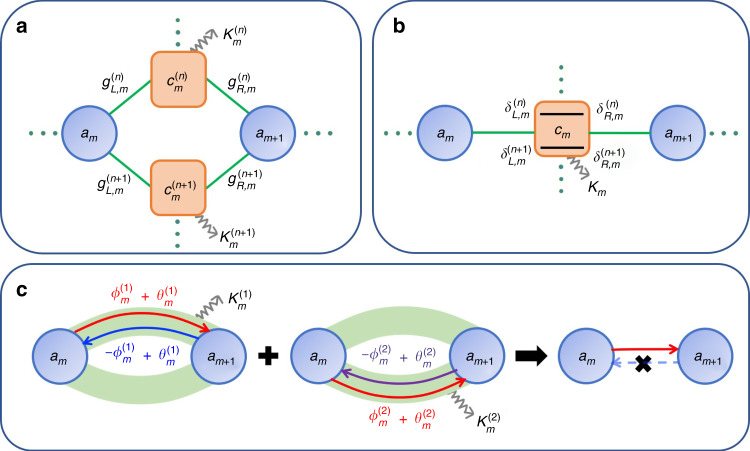
Schematic description of the scheme. **a** Sketch of a system with an array of main resonance modes $a_m$ connected via a series of connecting modes $c_m^{(n)}$. The loss of $c_m^{(n)}$ is represented by the decay rate $\kappa _m^{(n)}$. The coupling coefficient between *a*_*m*_ (*a*_*m+*1_) and $c_m^{(n)}$ is denoted by $g_{L,m}^{(n)}$ ($g_{R,m}^{(n)}$). **b** Sketch of a multichannel coupled system with synthetic frequency dimensions. Only one connecting mode *c*_*m*_ is used to connect *a*_*m*_ and *a*_*m+*1_, whereas the coupling between *a*_*m*_ (*a*_*m+*1_) and *c*_*m*_ has a series of detunings $\delta _{L,m}^{(n)}$ ($\delta _{R,m}^{(n)}$). **c** Illustration of multichannel interference for realizing nonreciprocity. For the first (second) coupling channel, the coherent coupling phase is $\phi _m^{(1)}$ ($\phi _m^{(2)}$) for the forward coupling and $- \phi _m^{(1)}$ ($- \phi _m^{(2)}$) for the backward coupling, whereas the loss phases are $\theta _m^{(1)}$ ($\theta _m^{(2)}$) for both the forward and backward couplings. By tuning the phases, constructive (destructive) interference can be achieved for the forward (backward) coupling. Thus, the forward coupling survives while the backward coupling vanishes

As illustrated in Fig. 1a, we consider a generic system in which an array of main resonance modes *a*_*m*_ (*m* = 1, 2, … *M*) are linked by a series of connecting modes $c_m^{(n)}\,(n = 1,2,...N)$ with decay rates $\kappa _m^{(n)}$. This model can be implemented in a variety of systems, such as optical cavities^41^, superconducting circuits^42^, mechanical resonators^43^, and atomic ensembles^44^. In the frame rotating at the input laser frequency *ω*_1_, the system Hamiltonian is given by ($\hbar = 1$)

1 $$\begin{array}{*{20}{l}} \hfill {H}={ - \mathop {\sum }\limits_{m = 1}^M {\mathrm{{\Delta}}}_ma_m^\dagger a_m - \mathop {\sum }\limits_{m = 1}^{M - 1} \mathop {\sum }\limits_{n = 1}^N {\Delta}_m^{\left( n \right)}c_m^{\left( n \right)\dagger }c_m^{\left( n \right)}}\\{ +\,\mathop {\sum }\limits_{m = 1}^{M - 1} \mathop {\sum }\limits_{n = 1}^N (g_{L,m}^{\left( n \right)}a_m^\dagger + g_{R,m}^{\left( n \right)}a_{m + 1}^\dagger )c_m^{\left( n \right)} + H.c.}\end{array}$$

where ${\Delta}_m \equiv \omega _{\mathrm{l}} - \omega _m$ and ${\mathrm{{\Delta}}}_m^{(n)} \equiv \omega _{\mathrm{l}} - \omega _m^{(n)}$ represent the detunings, with *ω*_*m*_ ($\omega _m^{(n)}$) being the resonance frequency of mode *a*_*m*_ ($c_m^{(n)}$) and $g_{L,m}^{(n)}$ ($g_{R,m}^{(n)}$) being the coupling coefficient between *a*_*m*_ (*a*_*m*+1_) and $c_m^{(n)}$. Here, the indices of the main (connecting) modes are denoted by subscripts (superscripts in parentheses) to avoid confusion.

In the above model, *N* connecting modes are required to realize $N$-channel coupling between *a*_*m*_ and *a*_*m*+1_. As sketched in Fig. 1b, this multichannel coupling can also be realized by using only one connecting mode *c*_*m*_ with synthetic frequency dimensions, where *N* pairs of coupling detunings $\delta _{L/R,m}^{(n)}$ play the role of *N* coupling channels. In this case, the system Hamiltonian is expressed as

$$H = - \mathop {\sum }\limits_{m = 1}^M {\mathrm{{\Delta}}}_ma_m^\dagger a_m - \mathop {\sum }\limits_{m = 1}^{M - 1} {\mathrm{{\Delta}}}_m^{\mathrm{c}}c_m^\dagger c_m + \mathop {\sum }\limits_{m = 1}^{M - 1} \mathop {\sum }\limits_{n = 1}^N \left[ \left(g_{L,m}^{(n)}e^{i\delta _{L,m}^{(n)}t}a_m^\dagger + g_{R,m}^{(n)}e^{i\delta _{R,m}^{(n)}t}a_{m + 1}^\dagger \right)c_m + H.c.\right]$$

where ${\Delta}_m^{\mathrm{c}} \equiv \omega _{\mathrm{l}} - \omega _m^{\mathrm{c}}$ is the detuning of the connecting mode *c*_*m*_. By expressing the connecting mode as $c_{m} = \mathop {\sum }\limits_{n = 1}^N (c_{L,m}^{(n)}e^{ - i\delta _{L,m}^{(n)}t} + c_{R,m}^{(n)}e^{ - i\delta _{R,m}^{(n)}t})$, where the $c_{L/R,m}^{(n)}$ are the components corresponding to the coupling detunings $\delta _{L/R,m}^{(n)}$, this Hamiltonian can ultimately be reduced to Eq. (1) under the rotating-wave approximation (see the Supplementary Information for details).

When the detunings ${\Delta}_m^{(n)}$ or decay rates $\kappa _m^{(n)}$ of the connecting modes are much larger than the coupling rates ($| {{\Delta}_m^{(n)} + i\kappa _m^{(n)}/2} | \gg | {g_{L/R,m}^{(n)}} |$), the connecting modes $c_m^{(n)}$ can be adiabatically eliminated^45,46^, leading to an effective non-Hermitian Hamiltonian $H_{{\mathrm{eff}}} = H_{{\mathrm{eff}}}^0 + H_{{\mathrm{eff}}}^{{\mathrm{int}}}$ (see the Supplementary Information for details). Here, $H_{{\mathrm{eff}}}^0 = - \mathop {\sum }\limits_{m = 1}^M ({\mathrm{{\Delta}}}_m + i\gamma _m/2 + {\Omega}_m)a_m^\dagger a_m$ is the free-energy term, with *γ*_*m*_ being the intrinsic linewidth of mode *a*_*m*_ and ${\it{{\Omega}}}_m = - \mathop {\sum}\nolimits_{n = 1}^N {\left[ {\left| {g_{L,m}^{(n)}} \right|^2/\left( {{\Delta}_m^{(n)} + i\kappa _m^{(n)}/2} \right) + \left| {g_{R,m - 1}^{(n)}} \right|^2/\left( {{\mathrm{{\Delta}}}_{m - 1}^{(n)} + i\kappa _{m - 1}^{(n)}/2} \right)} \right]}$ being the resonance shift and broadening; $H_{{\mathrm{eff}}}^{{\mathrm{int}}}$ is the interaction term, given by

2 $$\begin{array}{*{20}{r}} \hfill {H_{{\mathrm{eff}}}^{{\mathrm{int}}} = } & \hfill {\mathop {\sum }\limits_{m = 1}^{M - 1} (h_{m + 1,m}a_{m + 1}^\dagger a_m + h_{m,m + 1}a_m^\dagger a_{m + 1})}\\ \hfill {\,} & \hfill {h_{m + 1,m} = \mathop {\sum }\limits_{n = 1}^N G_m^{\left( n \right)}e^{ - i\phi _m^{\left( n \right)} - i\theta _m^{\left( n \right)}}}\\ \hfill {\,} & \hfill {h_{m,m + 1} = \mathop {\sum }\limits_{n = 1}^N G_m^{\left( n \right)}e^{i\phi _m^{\left( n \right)} - i\theta _m^{\left( n \right)}}}\end{array}$$

where *h*_*m*+1,*m*_ (*h*_*m*,*m*+1_) is the effective coupling coefficient for the forward (backward) direction between *a*_*m*_ and *a*_*m+*1_. It is seen that the total effective coupling coefficient is a sum of the effective coupling coefficients for each coupling channel. For the *n*-th channel, the amplitude of the effective coupling coefficient is $G_m^{(n)} \equiv \left| {g_{L,m}^{(n)}g_{R,m}^{(n)}} \right|/\sqrt {{\mathrm{{\Delta}}}_m^{(n)2} + \kappa _m^{(n)2}/4}$, whereas the phase factors include two components: $\phi _m^{(n)}$ and $\theta _m^{(n)}$. The first component, $\phi _m^{(n)} \equiv {\mathrm{arg}}\left( {g_{L,m}^{(n)}g_{R,m}^{(n) \ast }} \right)$, refers to the coherent coupling phase, which changes its sign when the coupling direction is reversed as a result of energy conservation. The second component, $\theta _m^{(n)} \equiv {\mathrm{arg}}({\Delta}_m^{(n)} + i\kappa _m^{(n)}/2)$, represents the phase lag induced by loss (the loss phase). It is noteworthy that this loss phase is determined only by the loss-detuning ratio, not by the coupling direction, as the losses play the same role for both the forward and backward couplings.

In the absence of losses, i.e., when $\kappa _m^{(n)} = 0$ and thus $\theta _m^{(n)} = 0$, *h*_*m*,*m*+1_ and *h*_*m*+1,*m*_ are complex conjugates, and $H_{{\mathrm{eff}}}^{{\mathrm{int}}}$ is Hermitian. In the presence of losses, the phase lag $\theta _m^{(n)}$ yields a non-Hermitian $H_{{\mathrm{eff}}}^{{\mathrm{int}}}$ with $h_{m,m + 1} \ne h_{m + 1,m}^ \ast$. When only one loss channel exists (*N* = 1), the effective coupling amplitudes for the forward and backward couplings are still the same, i.e., $\left| {h_{m,m + 1}} \right| = \left| {h_{m + 1,m}} \right|$, meaning that a nonreciprocal energy flow does not exist. This is consistent with the conclusion in the previous literature^2,3^, in which lossy systems are regarded as reciprocal in terms of restricted time-reversal symmetry. However, when more than one channel exists, the interference between different channels ultimately leads to unequal effective coupling amplitudes for the forward and backward couplings, i.e., $\left| {h_{m,m + 1}} \right| \ne \left| {h_{m + 1,m}} \right|$ for *N* ≥ 2. As depicted in Fig. 1c, the existence of the loss phase ensures that the interference properties are different for the forward and backward couplings. By tuning the phases, the forward coupling can be made to experience constructive interference, whereas the backward coupling will undergo destructive interference, leading to a nonzero forward coupling strength but a backward coupling strength of zero. This asymmetric coupling leads to an asymmetric scattering matrix (see the Supplementary Information for details), which indicates that the Lorentz reciprocity is broken^1^. Thus, nonreciprocity can be realized in a lossy system with multichannel interference. It is noteworthy that neither a magnetic field, the spatiotemporal modulation of permittivity, nor nonlinearity is required, and the nonreciprocity originates purely from the losses, which break the time-reversal symmetry.

Without loss of generality, in the following, we consider a two-channel situation (*N* = 2). To realize complete nonreciprocity, the amplitudes of the coupling coefficients for the two channels should be the same, i.e., $G_m^{(1)} = G_m^{(2)}$ (denoted by *G* in the following), so that complete destructive interference can be achieved. In this case, the forward and backward coupling coefficients are given by

3 $$h_ \rightleftarrows = 2Ge^{ \mp i\bar \phi - i\bar \theta }{\mathrm{cos}}\frac{{{\mathrm{{\Delta}}}\phi \pm {\mathrm{{\Delta}}}\theta }}{2}$$

where $h_ \to$ ($h_ \leftarrow$) is short for $h_{m + 1,m}$ ($h_{m,m + 1}$), ${\mathrm{{\Delta}}}\phi = \phi _m^{(2)} - \phi _m^{(1)}$ is the difference between the coherent coupling phases for the two channels, ${\Delta}\theta = \theta _m^{(2)} - \theta _m^{(1)}$ is the loss phase difference, and $\bar \phi = (\phi _m^{(1)} + \phi _m^{(2)})/2$ and $\bar \theta = (\theta _m^{(1)} + \theta _m^{(2)})/2$ are the corresponding average phases. Here, the subscripts “$m$” for the $m$-th main mode are omitted for convenience, as we mainly focus on the *M* = 2 case. It is clearly revealed that the phase difference leads to distinct interference patterns for the forward and backward couplings. The conditions for unidirectional nonreciprocal coupling are given by

4 $$\begin{array}{l}{\mathrm{{\Delta}}}\phi \mp {\mathrm{{\Delta}}}\theta = \pi + 2k\pi \\ \,\,{\Delta}\phi \ne p\pi ,{\mathrm{{\Delta}}}\theta \ne q\pi \end{array}$$

where *k*, *p*, and *q* are integers. Here, “−” corresponds to unidirectional forward coupling with $\left| {h_ \to } \right| \ne 0$ and $\left| {h_ \leftarrow } \right| = 0$, whereas “+” corresponds to unidirectional backward coupling with $\left| {h_ \to } \right| = 0$ and $\left| {h_ \leftarrow } \right| \ne 0$.

**Fig. 2:**
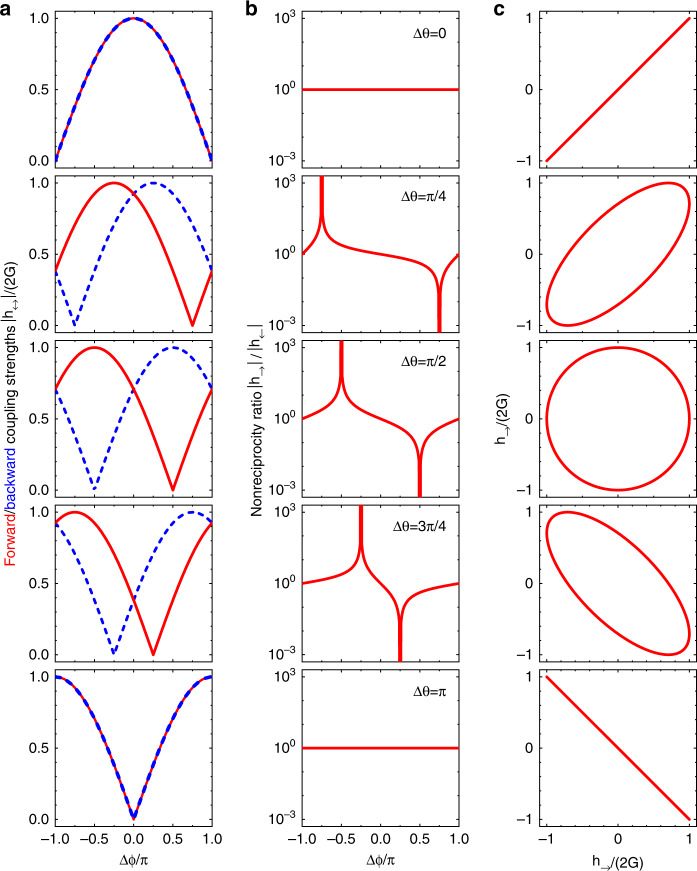
Forward and backward coupling strengths. **a** Amplitudes of the forward coupling $|h_ \to |$ (red solid curve) and the backward coupling $|h_ \leftarrow |$ (blue dashed curve) as functions of ${\Delta}\phi$. **b** Nonreciprocity ratio $|h_ \to |/|h_ \leftarrow |$ vs. ${\Delta}\phi$. **c** Parametric plots of $h_ \to$ and $h_ \leftarrow$ as functions of ${\Delta}\phi$. The parameters are ${\Delta}\theta = 0$ (first row), ${\Delta}\theta = \pi /4$ (second row), ${\Delta}\theta = \pi /2$ (third row), ${\Delta}\theta = 3\pi /4$ (fourth row), and ${\Delta}\theta = \pi$ (fifth row)

In Fig. 2a, we plot the amplitudes of the forward and backward couplings $\left| {h_ \rightleftarrows } \right|$ as functions of ${\Delta}\phi$ for various loss phase differences Δ*θ*. When Δ*θ* = 0 or *π*, the curves of $\left| {h_ \rightleftarrows } \right|$ coincide and thus, nonreciprocity is not achievable. However, for other values of Δ*θ*, the difference between $|h_ \to |$ and $|h_ \leftarrow |$ becomes significant. The curves of $|h_ \rightleftarrows |$ have the same lineshape, but the positions are shifted to the left (right) by Δ*θ*, showing that nonreciprocity can be realized. As depicted in Fig. 2b, the nonreciprocity ratio $|h_ \to |/|h_ \leftarrow |$ reaches a maximum/minimum when ${\Delta}\phi = \pm ({\Delta}\theta - \pi )$, in agreement with Eq. (4). For example, Δ*θ* = *π*/2 and ${\Delta}\phi = - \pi /2$ (*π*/*2*) lead to unidirectional forward (backward) coupling (third row of Fig. 2). The nonreciprocal properties can also be illustrated by the trajectories in the parameter space expanded by *h*_→_ and *h*_←_, as shown in Fig. 2c. The loss phase difference Δ*θ* exactly matches the relative phase lag between *h*_→_ and *h*_←_, where Δ*θ* = 0 and *π* correspond to linear trajectories without nonreciprocity and Δ*θ* = *π*/2 corresponds to a circular trajectory with the most prominent nonreciprocity, whereas for other values, the trajectories are ellipses with modest nonreciprocity.

Under the unidirectional forward coupling condition with ${\Delta}\phi = {\Delta}\theta + \pi + 2k\pi$, the amplitude of the forward coupling coefficient is given by

5 $$\left| {h_ \to } \right| = 2G\left| {{\mathrm{sin}}{\Delta}\theta } \right|$$

**Fig. 3:**
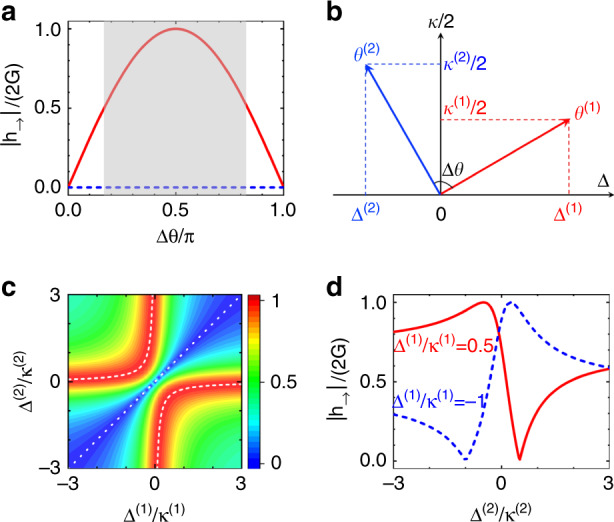
Optimization of the unidirectional forward coupling amplitude. **a** Unidirectional forward coupling amplitude $|h_ \to |$ (red solid curve) vs. ${\Delta}\theta$ under the condition of ${\Delta}\phi = \pm {\Delta}\theta + \pi + 2k\pi$. The backward coupling amplitude $|h_ \leftarrow |$ is 0 (blue dashed curve). **b** Sketch of the loss phase difference ${\Delta}\theta$ in the detuning-loss coordinate system. **c** Contour plot of $|h_ \to |$ as a function of ${\Delta}^{(1)}/\kappa ^{(1)}$ and ${\Delta}^{(2)}/\kappa ^{(2)}$. The white dashed curves correspond to the optimal conditions given in Eq. (3), whereas the white dotted curve represents the reciprocal case (${\Delta}^{(1)}\kappa ^{(2)} = {\Delta}^{(2)}\kappa ^{(1)}$). **d** $|h_ \to |$ vs. ${\Delta}^{(2)}/\kappa ^{(2)}$ for ${\Delta}^{(1)}/\kappa ^{(1)} = 0.5$ (red solid curve) and $- 1$ (blue dashed curve)

As illustrated in Fig. 3a, the loss phase difference Δ*θ* strongly determines the unidirectional coupling strength. The best performance is achievable for Δ*θ* = *π*/2, with the corresponding maximum being $|h_ \to |_{{\mathrm{max}}} = 2G$. It is noteworthy that the unidirectional coupling strength can remain large over a broad range of Δ*θ*, with the full width at half maximum being 2*π*/3 (shaded region). By expressing Δ*θ* in terms of the detunings and losses for the two coupling channels (Fig. 3b), we obtain ${\mathrm{tan}}\,{\mathrm{{\Delta}}}\theta = 2\left[ {{\mathrm{{\Delta}}}^{(1)}\kappa ^{(2)} - {\mathrm{{\Delta}}}^{(2)}\kappa ^{(1)}} \right]/\left[ {4{\mathrm{{\Delta}}}^{(1)}{\mathrm{{\Delta}}}^{(2)} + \kappa ^{(1)}\kappa ^{(2)}} \right]$. Thus, the optimal condition can be re-expressed as

6 $$\frac{{{\mathrm{{\Delta}}}^{(1)}}}{{\kappa ^{(1)}}}\frac{{{\mathrm{{\Delta}}}^{(2)}}}{{\kappa ^{(2)}}} = - \frac{1}{4}$$

whereas the condition for vanishing nonreciprocity is ${\mathrm{{\Delta}}}^{(1)}\kappa ^{(2)} = {\mathrm{{\Delta}}}^{(2)}\kappa ^{(1)}$. The contour map of $|h_ \to |$ as a function of ${\Delta}^{(1)}/\kappa ^{(1)}$ and ${\Delta}^{(2)}/\kappa ^{(2)}$ is plotted in Fig. 3c, and typical curves for $|h_ \to |$ as a function of ${\Delta}^{(2)}/\kappa ^{(2)}$ with a fixed ${\Delta}^{(1)}/\kappa ^{(1)}$ are plotted in Fig. 3d. These figures show that the parameter ranges for achieving a large $|h_ \to |$ are very broad. For a pure lossy system, two detunings with opposite signs are preferred, i.e., in one channel, the connecting mode is red detuned, whereas in the other channel the connecting mode should be blue detuned.

**Fig. 4:**
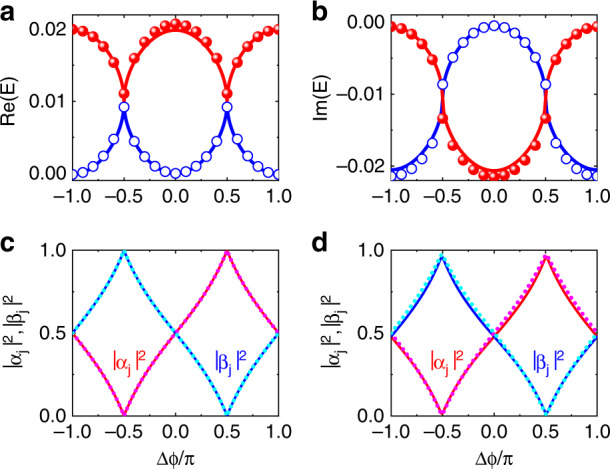
Eigenvalues and eigenmodes of the system. **a**, **b** Real (**a**) and imaginary (**b**) parts of the eigenvalues for the effective Hamiltonian [Eq. (2), curves] and the original Hamiltonian [Eq. (1), dots] as functions of ${\Delta}\phi$. **c**, **d** Expansion coefficients of the eigenmodes for the effective Hamiltonian (2) (**c**) and the original Hamiltonian (1) (**d**) as functions of ${\Delta}\phi$. The red solid and magenta dotted curves illustrate the distribution in mode *a*_*m*_ for each eigenmode ($|\alpha _{1,2}|^2$), and the blue solid and cyan dotted curves show the distribution in mode *a*_*m+*1_ for each eigenmode ($|\beta _{1,2}|^2$). The parameters are $\kappa ^{(1)} = \kappa ^{(2)} \equiv \kappa$, $\gamma _m/\kappa = \gamma _{m + 1}/\kappa = 10^{ - 3}$, ${\Delta}^{(1)}/\kappa = 80$, ${\mathrm{{\Delta}}}^{(2)}/\kappa = - \kappa /[4{\mathrm{{\Delta}}}^{(1)}]$, $g_L^{(1)}/\kappa = g_R^{(1)}/\kappa = 0.1\sqrt {|{\Delta}^{(1)}/\kappa + i/2|} ,\,g_L^{(2)}/\kappa = 0.1\sqrt {|{\Delta}^{(2)}/\kappa + i/2|}$, and $g_R^{(2)} = g_L^{(2)}e^{ - i{\mathrm{{\Delta}}}\phi }$

The nonreciprocity can also be reflected in the eigenvalues and eigenmodes of the system. In Fig. 4, we plot the real and imaginary parts of the energy eigenvalues and the expansion coefficients of the eigenmodes (defined as $e_j = \alpha _ja_m + \beta _ja_{m + 1}$, *j* = 1, 2) as functions of Δ*ϕ* for Δ*θ* = *π*/2 (corresponding to the third row in Fig. 2). For the reciprocal cases (Δ*ϕ* *=* *0*, ±*π*), the eigenvalues are split to the maximum extent, whereas the eigenmodes are equally weighted superpositions of *a*_*m*_ and *a*_*m+*1_. As the nonreciprocity ratio increases, for instance, Δ*ϕ* varies from 0 to ±*π*/2, the eigenvalue splitting becomes smaller, and the eigenmodes tend to be more localized in one of the main modes. For the completely nonreciprocal points, Δ*ϕ* *=* ±*π*/2, the eigenvalues become degenerate, accompanied by the coalescence of the eigenmodes, which are the features of exceptional points^47^. This is because the unidirectional nonreciprocal coupling causes one of the modes to be unstable and only one mode survives. For instance, Δ*ϕ* *=* −*π*/2 corresponds to a unidirectional forward coupling, meaning that the energy irreversibly flows from *a*_*m*_ to *a*_*m+*1_ and, thus, only *a*_*m+*1_ survives as the eigenmode ($|\beta _j| = 1$). It is worth noting that the results obtained by diagonalizing the effective Hamiltonian (2) with adiabatic elimination are consistent with those obtained from the original Hamiltonian (1), as shown in Fig. 4.

**Fig. 5:**
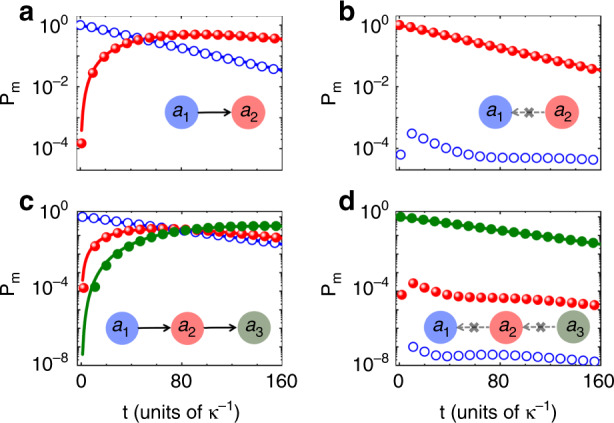
Unidirectional energy transmission. **a**, **b** Time evolution of the energy in the first main mode *a*_1_ (blue) and the second main mode *a*_2_ (red) for energy initially stored in the leftmost mode (**a**) and the rightmost mode (**b**). **c**, **d** Time evolution of the energy in *a*_1_ (blue), *a*_2_ (red), and *a*_3_ (black) for energy initially stored in the leftmost mode (**c**) and the rightmost mode (**d**). The curves are calculated from the effective Hamiltonian (2) and the dots are obtained from the original Hamiltonian (1). The insets illustrate the energy flow direction. The parameters are the same as in Fig. 4, with ${\Delta}\phi = - \pi /2$

A unidirectional nonreciprocal coupling directly gives rise to unidirectional energy transmission between the main resonance modes. In Fig. 5, we plot the typical results of unidirectional energy transmission for two (*M* = 2) and three (*M* = 3) main modes in the case of a forward unidirectional coupling ($|h_ \to | \ne 0$ and $|h_ \leftarrow | = 0$). It is revealed that forward transmission is allowed (Fig. 5a, c), whereas backward transmission is forbidden. The residual backward transmission, which originates from the imperfect adiabatic elimination of the connecting modes, is only 10^−4^ for *M* = 2 and $10^{ - 8}$ for *M* = 3 (Fig. 5b, d). The results calculated from the effective Hamiltonian (2) with adiabatic elimination (curves) agree well with those obtained from the original Hamiltonian (1) (dots).

## Discussion

Breaking Lorentz reciprocity is a very challenging task due to the time-reversal symmetry and linear nature of Maxwell’s equations, and at present, only three routes towards realizing nonreciprocity have been discovered, using a magnetic field, spatiotemporal modulation, or nonlinearity. Although loss breaks the time-reversal symmetry according to the traditional definition of time reversal, it is commonly believed that in classical electrodynamics, only restricted time reversal is valid. In this framework, lossy materials remain lossy under time-reversal transformation and restricted time-reversal symmetry still holds, which implies reciprocity^2,3^. Here we demonstrate that nonreciprocity can be achieved by making use of loss combined with multichannel interference. Due to the interference with different loss angles, the field ratios do not remain conserved under time-reversal transformation.

Although PT-symmetric systems also make use of losses^9,10,48^, it is clear that the nonreciprocity reported in previous PT-symmetric cavity schemes originates from the nonlinear gain saturation effect, i.e., the basic principle used to generate nonreciprocity is nonlinearity^9,10^. In our scheme, we focus on a pure lossy and linear system, in which neither gain nor nonlinearity is required and the fundamental aspect giving rise to nonlinearity is loss. It is noteworthy that loss is ubiquitous, whereas gain and nonlinearity are not common in optical systems. In addition, we clarify that the above results represent nonreciprocity but not simply asymmetrical power transmission, as the input and output channels both contain a single mode and the scattering matrix is asymmetric (see the Supplementary Information for details), indicating the breaking of Lorentz reciprocity. This can also be verified when we consider the implementation of our scheme by means of an experimentally feasible setup in which single-mode standing-wave photonic crystal cavities are connected by waveguides^49,50^ (see also the Supplementary Information for more details). The forward (backward) energy transmission coefficient can be defined as $T_ \to \equiv \left| {\left\langle {a_{m + 1}^{{\mathrm{out}}}/a_m^{{\mathrm{in}}}} \right\rangle } \right|^2\left( {T_ \leftarrow \equiv \left| {\left\langle {a_m^{{\mathrm{out}}}/a_{m + 1}^{{\mathrm{in}}}} \right\rangle } \right|^2} \right)$, which is equal to the modular square of the off-diagonal element $S_{m + 1,m}$ ($S_{m,m + 1}$) of the scattering matrix and proportional to the modular square of the forward (backward) coupling coefficient *h*_→_ (*h*_←_). Once the forward and backward coupling strengths are tuned to be unequal, the asymmetric scattering matrix leads to asymmetric forward and backward transmission coefficients, yielding nonreciprocity. Moreover, the unidirectional forward (backward) energy transmission coefficient can be maximized to 68.6% by optimizing the system parameters (the detailed derivations can be found in the Supplementary Information), corresponding to a 1.6 dB insertion loss.

In summary, we present the principle of loss-induced nonreciprocity, which is completely different from the existing principles relying on a magnetic field, spatiotemporal modulation, or nonlinearity. We design a coupled-mode model with a series of resonance modes interacting with each other via lossy connecting modes. A lossy mode possesses a phase lag induced by energy loss, which does not depend on the energy transmission direction. The interference between different coupling channels with different loss phases results in different coupling strengths for the forward and backward directions, yielding nonreciprocity. This property can be exactly tuned by matching the coherent coupling phases and loss phases, which depend on the ratio between the detuning and energy decay rate of the resonance modes. Our model is universal and can be applied to a variety of systems that can be described by resonance modes, such as optical cavities and waveguides, mechanical resonators, and superconducting circuits. Our work provides new opportunities for designing nonreciprocal optical devices and exploring topological properties such as the non-Hermitian skin effect^51^ without requiring a magnetic field, spatiotemporal modulation, nonlinearity, or other stringent conditions, and it may also inspire the further exploration of methods of turning harmful effects into resources.

## Supplementary information

Supplementary Information for “Loss induced nonreciprocity”
