# Supplementary material for: Loss-induced nonreciprocity
Source: Light Sci Appl. 2021 Feb 4;10:30. doi: 10.1038/s41377-021-00464-2 (PMC7862403; doi:10.1038/s41377-021-00464-2)
Supplement: Supplementary file 1 — Supplementary Information for “Loss induced nonreciprocity” [file 41377_2021_464_MOESM1_ESM.pdf]

Supplementary Information for "Loss induced nonreciprocity"

Xinyao Huang[1], Cuicui Lu[2], Chao Liang[1], Honggeng Tao[1], and Yong-Chun Liu[1,3*]

[1] *State Key Laboratory of Low-Dimensional Quantum Physics, Department of Physics, Tsinghua University, Beijing 100084, China*

[2] *Key Laboratory of Advanced Optoelectronic Quantum Architecture and Measurements of Ministry of Education, Beijing Key Laboratory of Nanophotonics and Ultrafine Optoelectronic Systems, School of Physics, Beijing Institute of Technology, Beijing 100081, China and*

[3] *Frontier Science Center for Quantum Information, Beijing 100084, China*

This Supplementary Information provides: (i) derivation of the effective Hamiltonian (Sec. I), (ii) derivation of multichannel coupling provided by the synthetic frequency dimension (Sec. II), (iii) derivation of unidirectional coupling condition (Sec. III), (iv) derivation of maximum coupling condition (Sec. IV), (v) additional results on unidirectional energy transmission (Sec. V), and (vi) discussion about single mode cavity-waveguide implementation (Sec. VI).

Contents

I. Deriviation of the effective Hamiltonian 1

II. Multichannel coupling provided by synthetic frequency dimension 3

III. Condition of unidirectional coupling 4

IV. Condition of maximum unidirectional coupling 5

V. Unidirectional energy transmission 6

VI. Single-mode cavity-waveguide implementation 7

References 9

I. DERIVIATION OF THE EFFECTIVE HAMILTONIAN

In this section, we present the details for the derivation of the effective Hamiltonian [Eq. (2)] given in the main text. Consider an array of main resonance modes a_m ($m = 1, 2, ...M$), which do not directly interact with each other. As sketched in Fig. 1 of the main text, for the m-th mode a_m and $(m+1)$-th mode a_{m+1}, they are connected by a series of connecting modes $c_m^{(n)}$ ($n = 1, 2, ...N$), corresponding to N-channel coupling. The system Hamiltonian is given by ($\hbar = 1$)

$$H = \sum_{m=1}^{M} \omega_m a_m^\dagger a_m + \sum_{m=1}^{M-1}\sum_{n=1}^{N} \omega_m^{(n)} c_m^{(n)\dagger} c_m^{(n)} + \sum_{m=1}^{M-1}\sum_{n=1}^{N} [(g_{L,m}^{(n)} a_m^\dagger + g_{R,m}^{(n)} a_{m+1}^\dagger) c_m^{(n)} + H.c.], \tag{S1}$$

where ω_m is the resonance frequency of main mode a_m, $\omega_m^{(n)}$ is the resonance frequency of connecting mode $c_m^{(n)}$, $g_{L,m}^{(n)}$ is the coupling coefficient between a_m and $c_m^{(n)}$, $g_{R,m}^{(n)}$ is the coupling coefficient between $c_m^{(n)}$ and a_{m+1}. Here the subscripts "L" is short for left and "R" is short for right. To avoid confusion, the indices of the main modes are all denoted by the subscripts, while the indices of the connecting modes are denoted by the superscript with parentheses. In the following, we will use these rules of notation unless otherwise specified.

In the reference frame rotating at the input laser frequency ω_l, the Hamiltonian reads

$$H = -\sum_{m=1}^{M} \Delta_m a_m^\dagger a_m - \sum_{m=1}^{M-1}\sum_{n=1}^{N} \Delta_m^{(n)} c_m^{(n)\dagger} c_m^{(n)} + \sum_{m=1}^{M-1}\sum_{n=1}^{N} [(g_{L,m}^{(n)} a_m^\dagger + g_{R,m}^{(n)} a_{m+1}^\dagger) c_m^{(n)} + H.c.], \tag{S2}$$

*Electronic address: ycliu@tsinghua.edu.cn

where we have applied the replacement $a_m \to a_m e^{-i\omega_l t}$ and $c_m^{(n)} \to c_m^{(n)} e^{-i\omega_l t}$. Here $\Delta_m = \omega_l - \omega_m$ and $\Delta_m^{(n)} = \omega_l - \omega_m^{(n)}$ are the laser detunings with respect to the resonance frequencies of the corresponding modes. Taking the losses and noise fluctuations into consideration, the Langevin equations can be written as

$$\frac{da_m}{dt} = (i\Delta_m - \frac{\gamma_m}{2})a_m - i\sum_{n=1}^{N}(g_{L,m}^{(n)}c_m^{(n)} + g_{R,m-1}^{(n)}c_{m-1}^{(n)}) + \sqrt{\gamma_m^{\mathrm{ex}}}a_m^{\mathrm{in}} + \sqrt{\gamma_m^0}f_m^{\mathrm{in}}, \tag{S3}$$

$$\frac{dc_m^{(n)}}{dt} = (i\Delta_m^{(n)} - \frac{\kappa_m^{(n)}}{2})c_m^{(n)} - i(g_{L,m}^{(n)*}a_m + g_{R,m}^{(n)*}a_{m+1}) + \sqrt{\kappa_m^{(n)}}c_m^{(n)\mathrm{in}}. \tag{S4}$$

Here γ_m is the energy decay rate of mode a_m, and $\kappa_m^{(n)}$ is the energy decay rate of mode $c_m^{(n)}$, while a_m^{in} and $c_m^{(n)\mathrm{in}}$ are the corresponding noise operators.

When the detunings $\Delta_m^{(n)}$ or decay rates $\kappa_m^{(n)}$ of the connecting modes are much larger than the coupling rates, i.e., $|\Delta_m^{(n)} + i\kappa_m^{(n)}/2| \gg (|g_{L,m}^{(n)}|, |g_{R,m}^{(n)}|)$, the connecting modes $c_m^{(n)}$ can be adiabatically eliminated. A simplified derivation is setting $dc_m^{(n)}/dt = 0$ and from Eq. (S4) we obtain

$$c_m^{(n)} = \frac{(g_{L,m}^{(n)*}a_m + g_{R,m}^{(n)*}a_{m+1}) + i\sqrt{\kappa_m^{(n)}}c_m^{(n)\mathrm{in}}}{\Delta_m^{(n)} + i\kappa_m^{(n)}/2}. \tag{S5}$$

Substituting Eq. (S5) into the Langevin equation of a_m [Eq. (S3)], we have

$$\frac{da_m}{dt} = (i\Delta_m - \frac{\gamma_m}{2} + i\Omega_m)a_m - i(h_{m,m+1}a_{m+1} + h_{m,m-1}a_{m-1}) + \xi_m^{\mathrm{in}}. \tag{S6}$$

Here $h_{m,m+1}$ and $h_{m,m-1}$ are the effective coupling coefficients between mode a_m and mode a_{m+1}, given by

$$h_{m,m+1} = \sum_{n=1}^{N}\frac{g_{L,m}^{(n)}g_{R,m}^{(n)*}}{\Delta_m^{(n)} + i\kappa_m^{(n)}/2}, \tag{S7}$$

$$h_{m,m-1} = \sum_{n=1}^{N}\frac{g_{L,m-1}^{(n)*}g_{R,m-1}^{(n)}}{\Delta_{m-1}^{(n)} + i\kappa_{m-1}^{(n)}/2}. \tag{S8}$$

Using the replacement $m \to m+1$ in Eq. (S8), we have

$$h_{m+1,m} = \sum_{n=1}^{N}\frac{g_{L,m}^{(n)*}g_{R,m}^{(n)}}{\Delta_m^{(n)} + i\kappa_m^{(n)}/2}. \tag{S9}$$

From Eq. (S6) we can find that $h_{m,m+1}$ determines the change of mode a_m induced by mode a_{m+1}, thus $h_{m,m+1}$ corresponds to the effective coupling coefficient for the backward direction, i.e., from mode a_{m+1} to mode a_m. Similarly, $h_{m,m-1}$ represents the effective coupling coefficient for the forward direction from mode a_{m-1} to mode a_m, and thus $h_{m+1,m}$ represents the effective forward coupling from mode a_m to mode a_{m+1}.

From Eqs. (S7) and (S9) we can find that it is more convenient to rewrite these complex coefficients using amplitudes and phases as

$$h_{m+1,m} = \sum_{n=1}^{N}G_m^{(n)}e^{-i\phi_m^{(n)} - i\theta_m^{(n)}}, \tag{S10}$$

$$h_{m,m+1} = \sum_{n=1}^{N}G_m^{(n)}e^{i\phi_m^{(n)} - i\theta_m^{(n)}}, \tag{S11}$$

$$G_m^{(n)} \equiv \frac{|g_{L,m}^{(n)}g_{R,m}^{(n)}|}{\sqrt{\Delta_m^{(n)2} + \kappa_m^{(n)2}/4}}, \tag{S12}$$

$$\phi_m^{(n)} \equiv \arg(g_{L,m}^{(n)}g_{R,m}^{(n)*}), \tag{S13}$$

$$\theta_m^{(n)} \equiv \arg(\Delta_m^{(n)} + i\frac{\kappa_m^{(n)}}{2}), \tag{S14}$$

where $G_m^{(n)}$ is the amplitude of the effective coupling coefficient for the n-th channel, $\phi_m^{(n)}$ is the coherent coupling phase, and $\theta_m^{(n)}$ is the loss-induced phase lag.

In Eq. (S6) Ω_m describes the the resonance shift and broadening for mode a_m, which is induced by the connecting modes, and the explicit expression is given by

$$\Omega_m = -\sum_{n=1}^{N}\left(\frac{|g_{L,m}^{(n)}|^2}{\Delta_m^{(n)} + i\kappa_m^{(n)}/2} + \frac{|g_{R,m-1}^{(n)}|^2}{\Delta_{m-1}^{(n)} + i\kappa_{m-1}^{(n)}/2}\right). \tag{S15}$$

In addition, the noise operator ξ_m^{in} in Eq. (S6) is given by

$$\xi_m^{\text{in}} = \sqrt{\gamma_m^{\text{ex}}}a_m^{\text{in}} + \sqrt{\gamma_m^0}f_m^{\text{in}} + \sum_{n=1}^{N}\left(\frac{g_{L,m}^{(n)}\sqrt{\kappa_m^{(n)}}c_m^{(n)\text{in}}}{\Delta_m^{(n)} + i\kappa_m^{(n)}/2} + \frac{g_{R,m-1}^{(n)}\sqrt{\kappa_{m-1}^{(n)}}c_{m-1}^{(n)\text{in}}}{\Delta_{m-1}^{(n)} + i\kappa_{m-1}^{(n)}/2}\right), \tag{S16}$$

where the damping rate associate with the input field a_m^{in} is γ_m^{ex}, and the intrinsic damping rate γ_m^0 with the noise f_m^{in}.

From Eq. (S6) we can also obtain the effective non-Hermitian Hamiltonian as

$$H_{\text{eff}} = H_{\text{eff}}^0 + H_{\text{eff}}^{\text{int}}, \tag{S17}$$

$$H_{\text{eff}}^0 = -\sum_{m=1}^{M}(\Delta_m + i\frac{\gamma_m}{2} + \Omega_m)a_m^\dagger a_m \tag{S18}$$

$$H_{\text{eff}}^{\text{int}} = \sum_{m=1}^{M-1}(h_{m,m+1}a_m^\dagger a_{m+1} + h_{m+1,m}a_{m+1}^\dagger a_m). \tag{S19}$$

Here Eq. (S19) together with Eqs. (S10) and (S11) correspond to Eq. (2) of the main text.

II. MULTICHANNEL COUPLING PROVIDED BY SYNTHETIC FREQUENCY DIMENSION

In Sec. I the N-channel coupling between a_m and a_{m+1} is realized by using N different connecting modes $c_m^{(n)}$ ($n = 1, 2, \ldots N$). In this section we present the derivation for realizing multichannel coupling by employing only one connecting mode c_m with synthetic frequency dimension. In this case the system Hamiltonian is given by

$$H = \sum_{m=1}^{M}\omega_m a_m^\dagger a_m + \sum_{m=1}^{M-1}\omega_m^{\text{c}}c_m^\dagger c_m + \sum_{m=1}^{M-1}[(g_{L,m}a_m^\dagger + g_{R,m}a_{m+1}^\dagger)c_m + H.c.], \tag{S20}$$

where ω_m^{c} is the resonance frequency of the connecting mode c_m. Here $g_{L,m}$ is the coupling coefficient between a_m and c_m, while $g_{R,m}$ is the coupling coefficient between c_m and a_{m+1}, which are assumed to have different frequency components with the expression described by

$$g_{L,m} = \sum_{n=1}^{N} g_{L,m}^{(n)}e^{i\delta_{L,m}^{(n)}t}, \tag{S21}$$

$$g_{R,m} = \sum_{n=1}^{N} g_{R,m}^{(n)}e^{i\delta_{R,m}^{(n)}t}. \tag{S22}$$

In the reference frame rotating at the input laser frequency ω_{l}, the Hamiltonian is given by

$$H = -\sum_{m=1}^{M}\Delta_m a_m^\dagger a_m - \sum_{m=1}^{M-1}\Delta_m^{\text{c}}c_m^\dagger c_m + \sum_{m=1}^{M-1}\sum_{n=1}^{N}[(g_{L,m}^{(n)}e^{i\delta_{L,m}^{(n)}t}a_m^\dagger + g_{R,m}^{(n)}e^{i\delta_{R,m}^{(n)}t}a_{m+1}^\dagger)c_m + H.c.], \tag{S23}$$

where $\Delta_m^{\text{c}} \equiv \omega_{\text{l}} - \omega_m^{\text{c}}$ is the laser detuning of with respect to the connecting mode c_m. The Langevin equations are given by

$$\frac{da_m}{dt} = (i\Delta_m - \frac{\gamma_m}{2})a_m - i\sum_{n=1}^{N}(g_{L,m}^{(n)}e^{i\delta_{L,m}^{(n)}t}c_m + g_{R,m-1}^{(n)}e^{i\delta_{R,m}^{(n)}t}c_{m-1}) + \sqrt{\gamma_m^{\text{ex}}}a_m^{\text{in}} + \sqrt{\gamma_m^0}f_m^{\text{in}}, \tag{S24}$$

$$\frac{dc_m}{dt} = (i\Delta_m^{\text{c}} - \frac{\kappa_m}{2})a_m - i\sum_{n=1}^{N}(g_{L,m}^{*}e^{-i\delta_{L,m}^{(n)}t}a_m + g_{R,m}^{*}(t)e^{-i\delta_{R,m}^{(n)}t}a_{m+1}) + \sqrt{\kappa_m}c_m^{\text{in}}, \tag{S25}$$

It is convenient to expand the connecting mode c_m into different frequency components as

$$c_m = \sum_{n=1}^{N} (c_{L,m}^{(n)} e^{-i\delta_{L,m}^{(n)} t} + c_{R,m}^{(n)} e^{-i\delta_{R,m}^{(n)} t}). \tag{S26}$$

Following the similar adiabatic elimination calculation in Sec. (I) with $dc_{L/R,m}^{(n)}/dt = 0$, we obtain

$$c_{L,m}^{(n)} = \frac{g_{L,m}^{(n)*} a_m + i\sqrt{\kappa_m} c_{L,m}^{(n)\text{in}}}{\Delta_m^{\text{c}} + \delta_{L,m}^{(n)} + i\frac{\kappa_m}{2}}, \tag{S27}$$

$$c_{R,m}^{(n)} = \frac{g_{R,m}^{(n)*} a_{m+1} + i\sqrt{\kappa_m} c_{R,m}^{(n)\text{in}}}{\Delta_m^{\text{c}} + \delta_{R,m}^{(n)} + i\frac{\kappa_m}{2}}. \tag{S28}$$

Under the condition $\delta_{L,m}^{(n)} = \delta_{R,m}^{(n)} \equiv \delta_m^{(n)}$, by applying the rotating-wave approximation and keeping only the resonant terms, we obtain

$$\frac{da_m}{dt} = (i\Delta_m - \frac{\gamma_m}{2} + i\Omega_m')a_m - i(h_{m,m+1}' a_{m+1} + h_{m,m-1}' a_{m-1}) + \xi_m'^{\text{in}}, \tag{S29}$$

which exactly has the same form as Eq. (S6), while the slightly different parameters are given by

$$h_{m,m+1}' = \sum_{n=1}^{N} \frac{g_{L,m}^{(n)} g_{R,m}^{(n)*}}{\delta_m^{(n)} + \Delta_m^{\text{c}} + i\kappa_m/2}, \tag{S30}$$

$$h_{m+1,m}' = \sum_{n=1}^{N} \frac{g_{L,m}^{(n)*} g_{R,m}^{(n)}}{\delta_m^{(n)} + \Delta_m^{\text{c}} + i\kappa_m/2}, \tag{S31}$$

$$\Omega_m' = \sum_{n=1}^{N} (\frac{|g_{L,m}^{(n)}|^2}{\delta_m^{(n)} + \Delta_m^{\text{c}} + i\kappa_m/2} + \frac{|g_{R,m-1}^{(n)}|^2}{\delta_{m-1}^{(n)} + \Delta_{m-1}^{\text{c}} + i\kappa_{m-1}/2}), \tag{S32}$$

$$\xi_m'^{\text{in}} = \sqrt{\gamma_m^{\text{ex}}} a_m^{\text{in}} + \sqrt{\gamma_m^0} f_m^{\text{in}} + \sum_{n=1}^{N} (\frac{g_{L,m}^{(n)} \sqrt{\kappa_m^{(n)}} c_m^{(n)\text{in}}}{\delta_m^{(n)} + \Delta_m^{\text{c}} + i\kappa_m/2} + \frac{g_{R,m-1}^{(n)} \sqrt{\kappa_{m-1}^{(n)}} c_{m-1}^{(n)\text{in}}}{\delta_{m-1}^{(n)} + \Delta_{m-1}^{\text{c}} + i\kappa_{m-1}/2}). \tag{S33}$$

Compared with the multimode connecting case in Sec. (I), we can find that there are only two major difference: (i) the detuning $\Delta_m^{(n)}$ is replaced by $\delta_m^{(n)} + \Delta_m^{\text{c}}$, (ii) the decay rate $\kappa_m^{(n)}$ is replaced by κ_m.

In this case the effective non-Hermitian Hamiltonian is given by

$$H_{\text{eff}} = -\sum_{m=1}^{M} (\Delta_m + i\frac{\gamma_m}{2} + \Omega_m') a_m^\dagger a_m + \sum_{m=1}^{M-1} (h_{m,m+1}' a_m^\dagger a_{m+1} + h_{m+1,m}' a_{m+1}^\dagger a_m), \tag{S34}$$

which also has the same form as Eq. (S19).

III. CONDITION OF UNIDIRECTIONAL COUPLING

In this section, we present the details on the condition of realizing unidirectional coupling. As discussed in the main text, unidirectional coupling requires one of the forward and backward coupling coefficient to be 0. This can be achieved when we introduce $N > 1$ loss channels to generate interference, so as to tune the amplitude of the the coupling coefficients. Considering the simplest case of $N = 2$, from Eqs. (S10) and (S11) we obtain

$$h_{m+1,m} = G_m^{(1)} e^{-i\phi_m^{(1)} - i\theta_m^{(1)}} + G_m^{(2)} e^{-i\phi_m^{(2)} - i\theta_m^{(2)}}, \tag{S35}$$

$$h_{m,m+1} = G_m^{(1)} e^{i\phi_m^{(1)} - i\theta_m^{(1)}} + G_m^{(2)} e^{i\phi_m^{(2)} - i\theta_m^{(2)}}, \tag{S36}$$

To realize complete nonreciprocity, i.e., $h_{m,m+1} = 0$ or $h_{m+1,m} = 0$, the amplitude of the coupling coefficients for the two channels should be the same, i.e., $G_m^{(1)} = G_m^{(2)} \equiv G_m$, which can be written more explicitly as

$$G_m = \frac{|g_{L,m}^{(1)} g_{R,m}^{(1)}|}{\sqrt{\Delta_m^{(1)2} + \kappa_m^{(1)2}/4}} = \frac{|g_{L,m}^{(2)} g_{R,m}^{(2)}|}{\sqrt{\Delta_m^{(2)2} + \kappa_m^{(2)2}/4}}. \tag{S37}$$

Then the coupling coefficients can be simplified as

$$h_{m+1,m} = 2G_m e^{-i\bar{\phi}_m - i\bar{\theta}_m} \cos\frac{\Delta\phi_m + \Delta\theta_m}{2}, \tag{S38}$$

$$h_{m,m+1} = 2G_m e^{i\bar{\phi}_m - i\bar{\theta}_m} \cos\frac{\Delta\phi_m - \Delta\theta_m}{2}, \tag{S39}$$

where $\Delta\phi_m = \phi_m^{(2)} - \phi_m^{(1)}$ is the difference of the coherent coupling phases for the two channels, $\Delta\theta_m = \theta_m^{(2)} - \theta_m^{(1)}$ is the loss phase difference, $\bar{\phi}_m = (\phi_m^{(1)} + \phi_m^{(2)})/2$ and $\bar{\theta}_m = (\theta_m^{(1)} + \theta_m^{(2)})/2$ are the corresponding average phases. The above equations corresponds to Eq. (3) of the main text. In this Supplemental Material the subscripts "m" for the m-th main mode are not omitted for the completeness.

The condition for unidirectional forward coupling is given by $h_{m,m+1} = 0$ and $h_{m+1,m} \neq 0$, from which we obtain

$$\Delta\phi_m - \Delta\theta_m = \pi + 2k\pi, \tag{S40}$$

$$\Delta\phi_m + \Delta\theta_m \neq \pi + 2k'\pi, \tag{S41}$$

which can be simplified as

$$\Delta\phi_m - \Delta\theta_m = \pi + 2k\pi, \tag{S42}$$

$$\Delta\phi_m \neq p\pi, \tag{S43}$$

$$\Delta\theta_m \neq q\pi, \tag{S44}$$

where k, k', p, q are integers. Similarly, the condition for unidirectional forward coupling is given by $h_{m+1,m} = 0$ and $h_{m,m+1} \neq 0$, with the results

$$\Delta\phi_m + \Delta\theta_m = \pi + 2k\pi, \tag{S45}$$

$$\Delta\phi_m \neq p\pi, \tag{S46}$$

$$\Delta\theta_m \neq q\pi. \tag{S47}$$

The above equations corresponds to Eq. (4) of the main text.

IV. CONDITION OF MAXIMUM UNIDIRECTIONAL COUPLING

To maximize the unidirectional coupling strength, we plug the condition for unidirectional forward coupling [Eq. (S42)] into Eq. (S38), which give rise to

$$|h_{m+1,m}| = 2G|\sin\Delta\theta|. \tag{S48}$$

This equation corresponds to Eq. (5) of the main text.

From Eqs. (S42) and (S48) we can find that the maximum unidirectional forward coupling is achievable for

$$\Delta\theta_m = \frac{\pi}{2} + j\pi, \tag{S49}$$

$$\Delta\phi_m = \frac{3\pi}{2} + (j + 2k)\pi, \tag{S50}$$

where j, k are integers. Without loss of generality, we can set $j = 1$ and $k = -1$, then the maximum unidirectional coupling corresponds to

$$\Delta\theta_m = \frac{\pi}{2}, \quad \Delta\phi_m = -\frac{\pi}{2}. \tag{S51}$$

To express the above optimal conditions in terms of direct system parameters, we can rewrite the loss phase difference using the detunings and decay rates. From the definition of loss phase $\theta_m^{(n)} = \arg(\Delta_m^{(n)} + i\frac{\kappa_m^{(n)}}{2})$, we obtain $\tan\theta_m^{(n)} = \kappa_m^{(n)}/(2\Delta_m^{(n)})$, and thereby

$$\tan\Delta\theta_m = \frac{2[\Delta_m^{(1)}\kappa_m^{(2)} - \Delta_m^{(2)}\kappa_m^{(1)}]}{4\Delta_m^{(1)}\Delta_m^{(2)} + \kappa_m^{(1)}\kappa_m^{(2)}}. \tag{S52}$$

Then the maximum unidirectional coupling condition $\Delta\theta_m = \pi/2 + j\pi$ is equivalent to

$$\frac{\Delta_m^{(1)}}{\kappa_m^{(1)}}\frac{\Delta_m^{(2)}}{\kappa_m^{(2)}} = -\frac{1}{4}, \tag{S53}$$

which corresponds to Eq. (6) of the main text.

From Eq. (S52) we can also find that $\Delta\theta_m = 0$ corresponds to $\Delta_m^{(1)}\kappa_m^{(2)} = \Delta_m^{(2)}\kappa_m^{(1)}$, and in this case the system is reciprocal.

V. UNIDIRECTIONAL ENERGY TRANSMISSION

In this section we discuss the unidirectional energy transmission for $M = 3$. In this case the effective Hamiltonian is given as

$$H_{eff} = -\sum_{m=1}^{3}(\Delta_m + i\frac{\gamma_m}{2} + \Omega_m)a_m^\dagger a_m + (h_{2,1}a_2^\dagger a_1 + h_{3,2}a_3^\dagger a_2 + h_{1,2}a_1^\dagger a_2 + h_{2,3}a_2^\dagger a_3). \tag{S54}$$

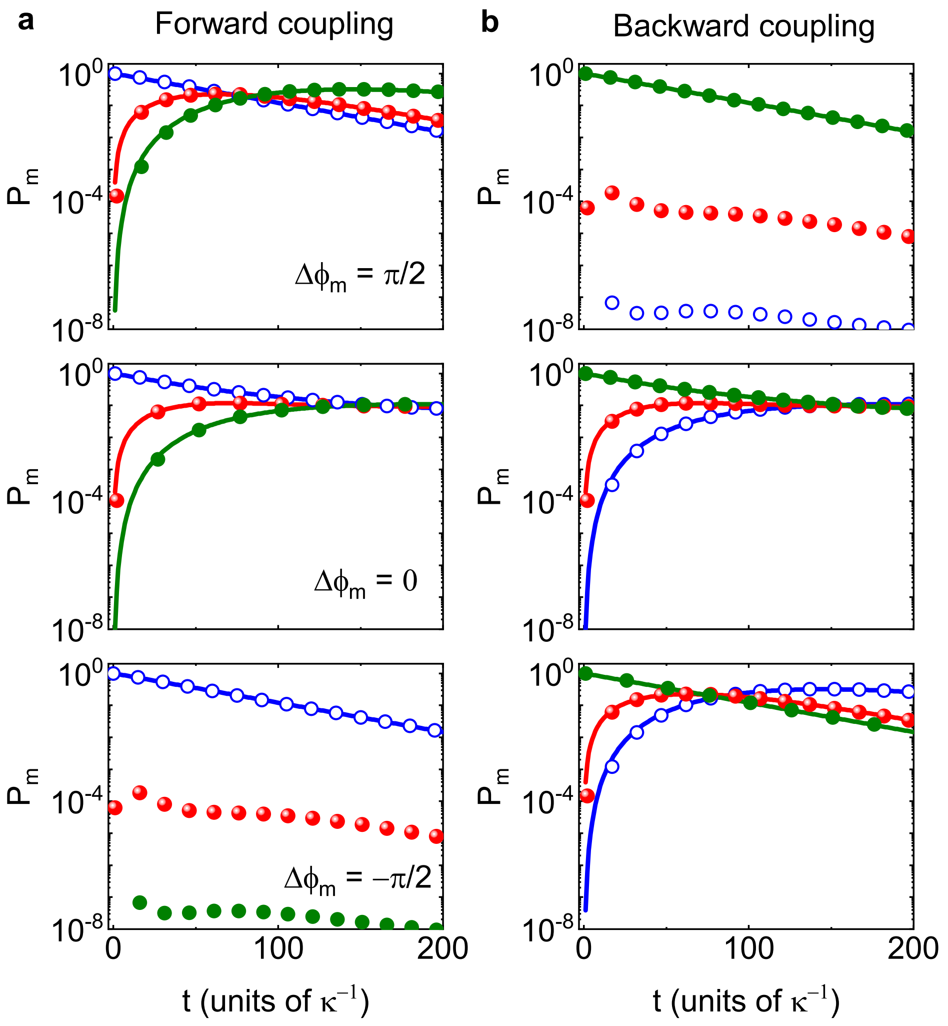

Fig. S1 Forward and backward Energy transmission. The time evolution of P_1 (blue), P_2 (red), and P_3 (black) when considering (**a**) $P_1(0) = 1$, $P_2(0) = P_3(0) = 0$ and (**b**) $P_1(0) = P_2(0) = 0$, $P_3(0) = 1$. The relevant parameters are: $\kappa_m^{(n)} = \kappa$, $\gamma_m = 10^{-3}\kappa$, $\Delta_m^{(1)} = 80\kappa$, $g_{L,m}^{(1)} = g_{R,m}^{(1)} = 0.1\sqrt{\kappa|\Delta_m^{(1)} + i\kappa/2|}$, $g_{L,m}^{(2)} = 0.1\sqrt{\kappa|\Delta_m^{(2)} + i\kappa/2|}$, $g_{R,m}^{(2)} = g_{L,m}^{(2)}e^{-i\Delta\phi_m}$ with $\Delta\phi_m = \pi/2$ (first line), $\Delta\phi_m = 0$ (second line), and $\Delta\phi_m = -\pi/2$ (third line).

Figure S1 plots the time evolution of the energy occupations in each mode P_m when considering three different sets of parameters: (i) $\Delta\phi_m = -\Delta\theta_m = -\pi/2$ (the first line), (ii) $\Delta\phi_m = 0, \Delta\theta_m = \pi/2$ (the second line), (iii) $\Delta\phi_m = \Delta\theta_m = \pi/2$ (the third line). In the first case, energy can transmit from a_1 to a_3 [Fig. S1a, the first line], while the opposite direction is forbidden [Fig. S1b, the first line]. Here the coupling coefficients are $h_{m,m+1} = 0, h_{m+1,m} = 2G_m$ in Eq. (S32), and the interaction terms become $(h_{2,1}a_2^\dagger a_1 + h_{3,2}a_3^\dagger a_2)$, which indicates that the energy can only transmit from a_1 to a_3. The opposite case is shown in the third line of Fig. S1, where the coupling coefficients are tuned as $h_{m+1,m} = 0, h_{m,m+1} = 2G_m$. From Eq. (S32) we can find the Hamiltonian only contains $(h_{1,2}a_1^\dagger a_2 + h_{2,3}a_2^\dagger a_3)$, meaning that energy can only transmit from a_3 to a_1. When considering the second case that $\Delta\phi_m = 0, \Delta\theta_m = \pi/2$, as shown in the second line of Fig. S1, unidirectional energy transmission cannot be achieved as $h_{m,m+1} = h_{m+1,m}$.

VI. SINGLE-MODE CAVITY-WAVEGUIDE IMPLEMENTATION

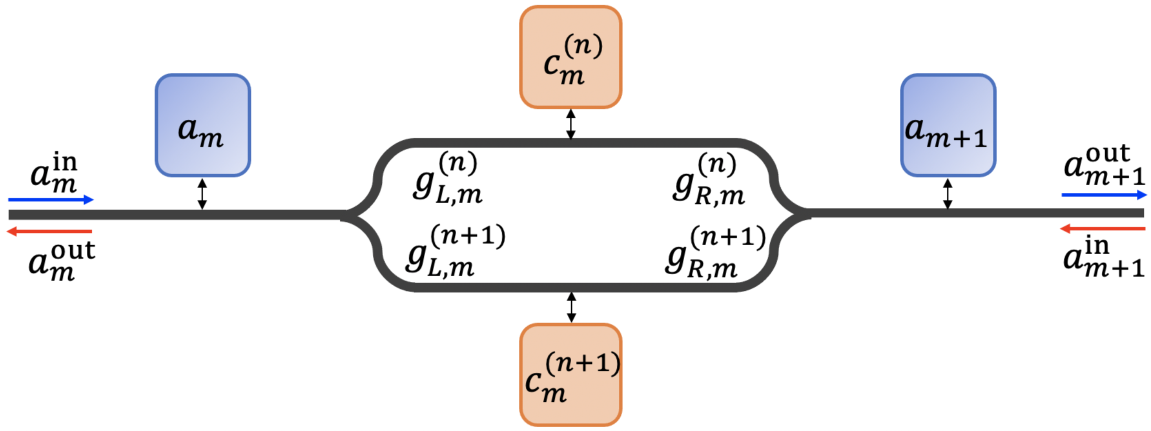

Fig. S2 Implementation of our scheme by using single-mode standing-wave photonic crystal cavities and waveguides. $g_{L/R,m}^{(n)}$ is the coupling coefficient between mode $a_{m,m+1}$ and mode $c_m^{(n)}$. The energy transmission in this system can either from a_m to a_{m+1} by taking a_m^{in} as the input and detecting a_{m+1}^{out} (direction shown by blue arrow), or from a_{m+1} to a_m when using a_{m+1}^{in} as the input and a_m^{out} as the output field (direction shown by red arrow), respectively.

As illustrated in Fig. S2, we consider a feasible implementation of our scheme by using a setup consisting of single-mode standing-wave photonic crystal cavities which are connected via waveguides [S1, S2]. The coupling between the cavities and the waveguides can induce indirect interaction between the cavity modes a_m and $c_m^{(n)}$ (with coupling coefficient $g_{L,m}^{(n)}$), a_m and $c_m^{(n+1)}$ (with coupling coefficient $g_{L,m}^{(n+1)}$), a_{m+1} and $c_m^{(n)}$ (with coupling coefficient $g_{R,m}^{(n)}$), a_{m+1} and $c_m^{(n+1)}$ (with coupling coefficient $g_{R,m}^{(n+1)}$). The phases of these coupling coefficients can be tuned by controlling the lengths of the coupling waveguides [S1, S2], thus the coherent coupling phases $\phi_m^{(n)} \equiv \arg(g_{L,m}^{(n)} g_{R,m}^{(n)*})$ are tunable. To achieve nonreciprocity, we can design the waveguide lengths so that the coherent coupling phases of the two channels satisfy $\Delta\phi_m = \phi_m^{(2)} - \phi_m^{(1)} \neq p\pi$, where p is an integer. On the other hand, the loss phases of the cavity modes can be individually controlled in each cavity, by tuning the resonance frequencies and the loss rates of the cavity modes, which are related to the cavity design. Therefore, the unidirectional nonreciprocal coupling condition (Eq. 4 of the main text) can be satisfied.

When adding the input field a_m^{in} (a_{m+1}^{in}) from the left (right) port, the output field a_{m+1}^{out} (a_m^{out}) can be detected on the right (left). The forward (backward) energy transmission can be described by defining the corresponding transmission coefficient $T_{m,m+1} = |\langle a_{m+1}^{\text{out}}/a_m^{\text{in}}\rangle|^2$ (forward, $\langle a_{m+1}^{\text{in}}\rangle = 0$) and $T_{m+1,m} = |\langle a_m^{\text{out}}/a_{m+1}^{\text{in}}\rangle|^2$ (backward, $\langle a_m^{\text{in}}\rangle = 0$), respectively.

To derive the expression of the transmission coefficients, we first rewrite the corresponding Langevin equations [Eq. (S6)] in the matrix form as

$$\frac{d\vec{v}}{dt} = M\vec{v} + \sqrt{\Gamma_{\text{ex}}}\vec{v}_{\text{in}} + \sqrt{\Gamma_0}\vec{f}_{\text{in}}, \tag{S55}$$

where $\vec{v}=(a_m,a_{m+1})^{\mathrm{T}}$, $\vec{v}_{\mathrm{in}}=(a_m^{\mathrm{in}},a_{m+1}^{\mathrm{in}})^{\mathrm{T}}$, and $\vec{f}_{\mathrm{in}}=(f_m^{in},f_{m+1}^{\mathrm{in}})^{\mathrm{T}}$. The coefficient matrix is

$$M=\begin{pmatrix} i(\Delta_m+\Omega_m)-\frac{\gamma_m}{2} & -ih_{m,m+1} \\ -ih_{m+1,m} & i(\Delta_{m+1}+\Omega_{m+1})-\frac{\gamma_{m+1}}{2}\end{pmatrix}, \tag{S56}$$

the damping rate associated with the input coupling is $\sqrt{\Gamma_{\mathrm{ex}}}=\mathrm{Diag}[\sqrt{\gamma_m^{\mathrm{ex}}},\sqrt{\gamma_{m+1}^{\mathrm{ex}}}]$, and the intrinsic damping rate is $\sqrt{\Gamma_0}=\mathrm{Diag}[\sqrt{\gamma_m^0},\sqrt{\gamma_{m+1}^0}]$.

We solve the Langevin equations [Eq. (S55)] in the frequency domain by transforming $\vec{v}(t)=\int d\omega e^{-i\omega t}\vec{v}(\omega)/2\pi$. Ignoring the intrinsic damping rate γ_m^0 of the mode a_m (i.e., $\gamma_m\approx\gamma_m^{\mathrm{ex}}$) and plugging the solution into the input-output relation $\vec{v}_{\mathrm{out}}(\omega)=\vec{v}_{\mathrm{in}}(\omega)-\sqrt{\Gamma_{\mathrm{ex}}}\vec{v}(\omega)$, where $\vec{v}_{\mathrm{out}}=(a_m^{\mathrm{out}},a_{m+1}^{\mathrm{out}})^{\mathrm{T}}$ is the output operator, we can obtain

$$\vec{v}_{\mathrm{out}}(\omega)=S(\omega)\vec{v}_{\mathrm{in}}(\omega). \tag{S57}$$

The scattering matrix $S(\omega)$ can be written as

$$S(\omega)=I+\sqrt{\Gamma_{\mathrm{ex}}}(i\omega+M)^{-1}\sqrt{\Gamma_{\mathrm{ex}}}, \tag{S58}$$

where I is the identity matrix. At the resonance point $\omega=0$, the off-diagonal elements of the scattering matrix S are derived as

$$S_{m,m+1}=\frac{i\sqrt{\gamma_m^{\mathrm{ex}}\gamma_{m+1}^{\mathrm{ex}}}h_{m,m+1}}{h_{m,m+1}h_{m+1,m}+(\gamma_m-2i(\Delta_m+\Omega_m)(\gamma_{m+1}-2i(\Delta_{m+1}+\Omega_{m+1}))/4}, \tag{S59}$$

$$S_{m+1,m}=\frac{i\sqrt{\gamma_m^{\mathrm{ex}}\gamma_{m+1}^{\mathrm{ex}}}h_{m+1,m}}{h_{m,m+1}h_{m+1,m}+(\gamma_m-2i(\Delta_m+\Omega_m)(\gamma_{m+1}-2i(\Delta_{m+1}+\Omega_{m+1}))/4}. \tag{S60}$$

Note that the off-diagonal elements of the scattering matrix ($S_{m,m+1}$ and $S_{m+1,m}$) are determined by the effective coupling coefficients $h_{m,m+1}$ and $h_{m+1,m}$. Once $|h_{m,m+1}|\neq|h_{m+1,m}|$ is tuned in our model, we can obtain asymmetric scattering matrix ($S_{m,m+1}\neq S_{m+1,m}$), i.e., the break of the Lorentz reciprocity. Applying Eqs. (S57)-(S60) to the transmission coefficient, we can find the forward transmission coefficient

$$T_{\rightarrow}\equiv|\langle\frac{a_{m+1}^{\mathrm{out}}}{a_m^{\mathrm{in}}}\rangle|^2=|S_{m+1,m}|^2=\frac{\gamma_m^{\mathrm{ex}}\gamma_{m+1}^{\mathrm{ex}}|h_{m+1,m}|^2}{|h_{m,m+1}h_{m+1,m}+(\gamma_m-2i(\Delta_m+\Omega_m)(\gamma_{m+1}-2i(\Delta_{m+1}+\Omega_{m+1}))/4|^2}, \tag{S61}$$

and the backward transmission coefficient is given as

$$T_{\leftarrow}\equiv|\langle\frac{a_m^{\mathrm{out}}}{a_{m+1}^{\mathrm{in}}}\rangle|^2=|S_{m,m+1}|^2=\frac{\gamma_m^{\mathrm{ex}}\gamma_{m+1}^{\mathrm{ex}}|h_{m,m+1}|^2}{|h_{m,m+1}h_{m+1,m}+(\gamma_m-2i(\Delta_m+\Omega_m)(\gamma_{m+1}-2i(\Delta_{m+1}+\Omega_{m+1}))/4|^2}. \tag{S62}$$

Obviously, we can find $T_{\rightarrow}\neq 0$ while $T_{\leftarrow}=0$ for unidirectional forward coupling ($h_{m,m+1}=0$). In the case of unidirectional backward coupling ($h_{m+1,m}=0$), the backward transmission coefficient $T_{\leftarrow}\neq 0$ while $T_{\rightarrow}=0$. So that unidirectional energy transmission also provides an efficient evidence of the nonreciprocity generation in our scheme.

Next we show how to maximize the unidirectional energy transmission coefficients. For unidirectional forward energy transmission ($|h_{m,m+1}|=0$), the coefficient is given by

$$T_{\rightarrow}=\frac{16\gamma^2|h_{m+1,m}|^2}{|(\gamma-2i\Omega_m)(\gamma-2i\Omega_{m+1})|^2}, \tag{S63}$$

where we have taken the assumption $\Delta_m=0$, $\gamma_m\approx\gamma_m^{\mathrm{ex}}\equiv\gamma$ for simplicity. Considering the case that the mode number is $M=N=2$, the resonance shift and broadening of mode a_m and a_{m+1} can be written as

$$\Omega_m=-\sum_{n=1}^{2}\frac{|g_{L,m}^{(n)}|^2e^{-i\theta_m^{(n)}}}{|\Delta_m^{(n)}+i\kappa_m^{(n)}/2|}, \tag{S64}$$

$$\Omega_{m+1}=-\sum_{n=1}^{2}\frac{|g_{R,m}^{(n)}|^2e^{-i\theta_m^{(n)}}}{|\Delta_m^{(n)}+i\kappa_m^{(n)}/2|}. \tag{S65}$$

Choosing $|g_{L,m}^{(n)}|=|g_{R,m}^{(n)}|$ as the optimal condition (by numerical optimization of the transmission coefficients), we can find

$$\Omega_m=\Omega_{m+1}=-Ge^{-i\theta_m^{(1)}}(1+e^{-i\Delta\theta}). \tag{S66}$$

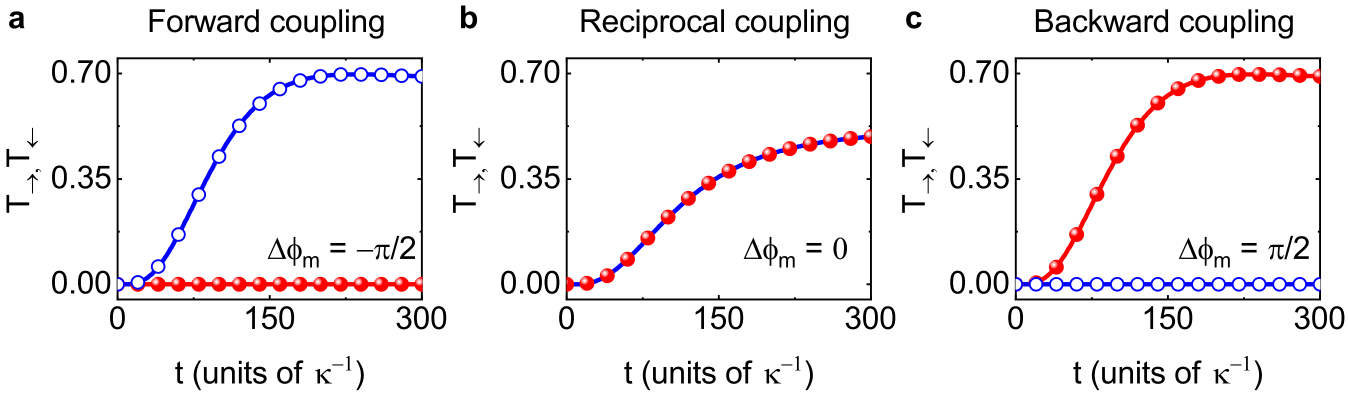

Fig. S3 Forward ($T_{\rightarrow}$) and backward ($T_{\leftarrow}$) transmission coefficients. The time evolution of $T_{\rightarrow}$ (blue), $T_{\leftarrow}$ (red) when considering three different cases: (a) $\Delta\phi_m = -\pi/2$, (b) $\Delta\phi_m = 0$, and (c) $\Delta\phi_m = \pi/2$. The relevant parameters are: $\kappa_m^{(n)} = \kappa$, $\gamma_m^{\mathrm{ex}} = \gamma_m$, $\gamma_m = 0.02\sqrt{2}\kappa$, $\Delta_m^{(1)} = 80\kappa$, $g_{L,m}^{(1)} = g_{R,m}^{(1)} = 0.1\sqrt{\kappa|\Delta_m^{(1)} + i\kappa/2|}$, $g_{L,m}^{(2)} = 0.1\sqrt{\kappa|\Delta_m^{(2)} + i\kappa/2|}$, $g_{R,m}^{(2)} = g_{L,m}^{(2)} e^{-i\Delta\phi_m}$. The results obtained from the effective and original Hamiltonian [Eq. (1) and (2) in the main text] are illustrated by curves and dots, respectively.

As $|h_{m+1,m}|_{\max} = 2G$ when $\Delta\theta = -\Delta\phi = \pi/2$, we can simplify the unidirectional forward energy transmission coefficient as

$$T_{\rightarrow} = \left(\frac{8\gamma G}{(\gamma + 2\sqrt{2}G\cos(\theta_m^{(1)} - \pi/4))^2 + 8G^2\sin^2(\theta_m^{(1)} - \pi/4)}\right)^2, \tag{S67}$$

where $\theta_m^{(1)} = \arg(\Delta_m^{(1)} + i\kappa_m^{(1)}/2)$. Hence we can obtain the maximized unidirectional forward energy transmission coefficient is $T_{\rightarrow,\max} = 4(3 - 2\sqrt{2}) \approx 0.686$ when optimizing the parameters as $\gamma = 2\sqrt{2}G$ and in the limit of very large $\Delta_m^{(1)}$. The same maximum can be obtained for the unidirectional backward energy transmission coefficient $T_{\leftarrow,\max} = T_{\rightarrow,\max} \approx 0.686$. In conclusion, the lost input energy can be minimized to 31.4% in the unidirectional energy transmission via our scheme.

To demonstrate the unidirectional energy transmission, we calculate the transmission coefficients for $M = 2$ under three different sets of system parameters: (a) $-\Delta\phi = \Delta\theta = \pi/2$, (b) $\Delta\phi = 0, \Delta\theta = \pi/2$, (c) $\Delta\phi = \Delta\theta = \pi/2$. In the first case[Fig. S3(a)], the forward unidirectional coupling ($|h_{m+1,m}| \neq 0$ and $|h_{m,m+1}| = 0$) allows the energy to be transmitted from a_m to a_{m+1}, while the opposite direction is forbidden. When adding the input field, it corresponds to nonzero forward transmission coefficient $T_{\rightarrow} \neq 0$ (blue) and zero backward transmission coefficient $T_{\leftarrow} \approx 0$ (red). The opposite situation is shown in Fig. S3(c), where the backward unidirectional coupling ($|h_{m,m+1}| \neq 0$ and $|h_{m+1,m}| = 0$) is engineered to make the energy only flow in the backward direction, so that the backward transmission coefficient $T_{\leftarrow} \neq 0$ (red), while the forward transmission coefficient $T_{\rightarrow} = 0$ (blue). When both the forward and backward coupling exist in the system ($|h_{m+1,m}| = |h_{m,m+1}| \neq 0$) [Fig. S3(b)], unidirectional energy transmission cannot be achieved, which corresponds to $T_{\rightarrow} = T_{\leftarrow} \neq 0$.

[S1] Sato, Y. et al. Strong coupling between distant photonic nanocavities and its dynamic control. *Nat. Photon.* **6**, 56-61 (2012).

[S2] Xiao, Y.-F. et al. Asymmetric Fano resonance analysis in indirectly coupled microresonators. *Phys. Rev. A* **82**, 065804 (2010).
